# Effective management of district-level malaria control and elimination: implementing quality and participative process improvements

**DOI:** 10.1186/s12889-021-12322-2

**Published:** 2022-01-20

**Authors:** Bruce Agins, Peter Case, Daniel Chandramohan, Ingrid Chen, Rudo Chikodzore, Precious Chitapi, Amanda Chung, Roly Gosling, Jonathan Gosling, Matsiliso Gumbi, Daniel Ikeda, Munashe Madinga, Peliwe Mnguni, Joseph Murungu, Cara Smith Gueye, Jim Tulloch, Greyling Viljoen, Leadership and Engagement for improved Accountability and Delivery of Services Framework Development Group, Bruce Agins, Peter Case, Daniel Chandramohan, Ingrid Chen, Rudo Chikodzore, Precious Chitapi, Amanda Chung, Roly Gosling, Jonathan Gosling, Matsiliso Gumbi, Daniel Ikeda, Munashe Madinga, Peliwe Mnguni, Joseph Murungu, Cara Smith Gueye, Jim Tulloch, Greyling Viljoen

**Affiliations:** 1 HEALTHQUAL, Institute of Global Health Sciences, University of California San Francisco, 550 16th Street, San Francisco, CA 94158 USA; 2 Department of Epidemiology and Biostatistics, University of California San Francisco, 550 16th Street, San Francisco, CA 94158 USA; 3 Bristol Business School, University of West of England, Frenchay Campus, Coldharbour Lane, Bristol, BS16 1QY UK; 4 College of Business, Law & Governance, James Cook University, Douglas, Australia; 5 Department of Disease Control, London School of Hygiene and Tropical Medicine, Keppel Street, London, WC1E 7HT UK; 6 Malaria Elimination Initiative, University of California San Francisco, 550 16th Street, San Francisco, CA 94158 USA; 7 Ministry of Health and Child Care Matabeleland South Province, New Government Complex, Third Ave, Gwanda, Zimbabwe; 8 Precious Innovations, 11 Dougal Rd, The Grange, Harare, Zimbabwe; 9 University of Exeter Business School, Rennes Dr, Exeter, EX4 4PU UK; 10 Ditsong Museums of South Africa, 70 WF Nkomo St, Pretoria, South Africa; 11 Clinton Health Access Initiative, Mount Pleasant, Harare, Zimbabwe; 12 Gaduate School of Business Leadership, University of South Africa, Preller St, Muckleneuk, Pretoria, South Africa; 13 Independent Consultant, GPO Box 1566, Adelaide, 5001 South Australia; 14 Independent Consultant, 342 Albert Street, Waterkloof, Pretoria South Africa

## Abstract

Although it is widely recognized that strong program management is essential to achieving better health outcomes, this priority is not recognized in malaria programmatic practices. Increased management precision offers the opportunity to improve the effectiveness of malaria interventions, overcoming operational barriers to intervention coverage and accelerating the path to elimination. Here we propose a combined approach involving quality improvement, quality management, and participative process improvement, which we refer to as Combined Quality and Process Improvement (CQPI), to improve upon malaria program management. We draw on evidence from other areas of public health, as well as pilot implementation studies in Eswatini, Namibia and Zimbabwe to support the proposal. Summaries of the methodological approaches employed in the pilot studies, overview of activities and an outline of lessons learned from the implementation of CQPI are provided. Our findings suggest that a malaria management strategy that prioritizes quality and participative process improvements at the district-level can strengthen teamwork and communication while enabling the empowerment of subnational staff to solve service delivery challenges. Despite the promise of CQPI, however, policy makers and donors are not aware of its potential. Investments are therefore needed to allow CQPI to come to fruition.

## Background

Operational issues such as delivery and management are major challenges across health systems worldwide [1]. Although these challenges compromise the efficiency and effectiveness of health systems, often preventing those in need from accessing quality care, program management is perhaps one of the most neglected areas in public health. This is especially true for malaria, with strong program management being an essential component for malaria control, elimination and eradication [2].

Delivery is especially important for malaria, as the most important interventions are based on the distribution of vector control interventions into affected communities, such as long-lasting insecticide-treated bed nets or indoor residual spraying of insecticides to the walls of houses [3]. The delivery of these interventions is rife with operational challenges, as is the provision of effective diagnosis, testing, and treatment of malaria at the community level. As countries approach malaria elimination, delivery of interventions require greater precision in time and space to targeted and often difficult to reach populations that need specifically tailored malaria control strategies.

How can the delivery of malaria interventions be improved, particularly as countries approach national and subnational malaria elimination? A review in 2015 revealed that program management improvement methods used outside of the health sector could provide numerous gains to provision of health and particularly malaria [4]. In this paper we highlight the value of three approaches: first, the use of standard quality improvement (QI), second, quality management (QM); and third, the use of Participative Process Improvement (PPI). QI and QM comprise techniques where people within the health systems are asked to seek improvement of a pre-defined problem, whilst PPI is a bottom-up approach where the problems chosen for improvement are defined by the people delivering the interventions (Table 1) [18, 19].

**Table 1:** Definitions and descriptions of combined quality and process improvement, quality improvement, quality management, and participative process improvement

Term	Definition
Combined Quality and Process Improvement (CQPI)	An approach that involves simultaneous implementation of three synergistic approaches to process improvement: Quality Improvement, Quality Management, and Participative Process Improvement.
Quality Improvement (QI)	Quality Improvement in this article to refer to a generic set of principles: systems-thinking which includes formal root cause analysis (QI toolbox); understanding variation; continuous cycles of measurement and improvement; testing of changes (Plan-Do-Study-Act); peer learning, teamwork, and involving consumers. Of these, the first three are the most essential [5].
Quality Management (QM)	QM relates to a HEALTHQUAL framework that has evolved and been trialed over time [6–8]. Elements include: 1) leadership and governance; 2) a formal QM plan; 3) organizational infrastructure, including a technical working group or committee; 4) a performance measurement system with specific indicators; 5) procedures for implementing and sustaining continuous QI activities; 6) workforce capacity building of QI capabilities; 7) patient/community involvement; 8) knowledge management; and 9) outcomes assessment.
Participative Process Improvement (PPI)	Participative Process Improvement, as referred to in this article, also known as participative action research [4, 9] is informed by aspects of generic QI and the HEALTHQUAL framework, but comprises a specific set of interventions designed to enhance healthcare service delivery and organizational effectiveness. Where organizing is seen as a process that requires continuous and numerous activities, PPI enhances capacity for crucial aspects of human relations and activities – typically the softer aspects that rely on such qualities as listening, respect, reflection, and adapting to ‘political’ realities [10–12]. PPI interventions often include structured techniques such as those included in QI and QM, along with others explicitly designed to evince insight from frontline and community-based stakeholders [13–15]; to engage line managers in responding to these insights; and to embed accountability for change at all levels of the system. Examples are a) peer-led problem solving, b) attentive listening, c) process mapping, and d) assessment of inter-group dynamics. PPI is most effective when a ‘system in the room’ approach is adopted. The term ‘system in the room’ is taken from the field of psychosocial studies and organizational dynamics [16, 17]. This entails replicating the programme/service delivery system as fully as possible in a shared workshop setting (e.g., a conference room). Representation includes not only the healthcare professionals at district level who are responsible for service provision, but also more senior staff and resource holders from provincial and ministry levels as well as community decision-makers and intended beneficiaries. Full representation enables sharing of perspectives and challenges from across the system and helps inform collaborative generation of challenges, synchronized solutions and collective support for those whose role it is to implement frontline solutions that involve changes in processes and procedures. Ensuring that those with the seniority to authorize and resource changes is critically important to the process.
Leadership and Engagement for improved Accountability and Delivery of Services Framework (LEAD Framework)	A practical tool to support the implementation of CQPI for health program use. The framework and supporting documents can be found at http://www.shrinkingthemalariamap.org/tool/leadership-engagement-improved-accountability-delivery-services-framework-lead

As they are synergistic, we suggest that the three approaches be combined to form one composite approach to malaria program management improvement. For convenience, we refer to the integrated approach as Combined Quality and Process Improvement (CQPI). We propose CQPI as a promising means of overcoming operational challenges to malaria control and elimination, with recent evidence suggesting CQPI can make a significant impact when focused at the district-level [20, 21]. Building upon the authors growing experience in implementing CQPI for malaria control and elimination, the team have developed the Leadership and Engagement for improved Accountability and Delivery of Services Framework (LEAD Framework) that provides detailed instructions to support program level implementation of CQPI [22].

## Main text

### Improving program management practices for malaria elimination

CQPI offers a new area of focus for malaria control and elimination programming that can substantially improve the quality and precision of intervention delivery. The approach incorporates rigorous methods for monitoring and evaluating organizational performance where challenges to implementation occur, and has been shown to improve user satisfaction and staff motivation while reducing consequences associated with inappropriate clinical decisions [6, 13, 23]. Most widely demonstrated to be useful in Human Immunodeficiency Virus (HIV) control programs, QI and QM are now being recognized for their effectiveness and impact by Ministries of Health, and are also being applied in maternal, newborn, and child health, and tuberculosis programs [14]. Within malaria programs, QI and QM are currently limited to quality assurance schemes for diagnostics, medicines, and occasionally for case management, typically in donor funded settings, but with tremendous potential to grow [14, 20].

For malaria, the addition of PPI to QI and QM also offers specific added value in addressing three major operational challenges to malaria elimination. First, in settings preparing for malaria elimination, those increasingly more at risk of malaria often have the weakest access to the health system, posing operational challenges to the effective and efficient delivery of services [15]. Second, malaria epidemiology becomes increasingly site specific, requiring tailored solutions that are best solved locally with input from frontline staff and communities. Third, funding for malaria tends to drop as it becomes less of a national priority, with staff often being required to deliver services to multiple health programs. Staff motivation can suffer as they typically do not have access to training or mentorship in time, resource or quality management; three challenges currently resulting from the top-down delivery of interventions with minimal input from affected communities [9]. PPI, which is designed to harness insight from local stakeholders, holds particular value for confronting these challenges.

CQPI therefore offers the potential to foster rigorous attention to vector data, urgent case management and response, and inventory control necessary for preventing transmission while cultivating qualities such as inventiveness, proactivity, accountability, mutual trust and confidence, all of which enhance staff motivation.

While program management techniques are most often applied at the health facility level, we recommend that CQPI for malaria control and elimination be applied through a subnational approach involving the interaction of district and regional level teams with facility staff to enable district-level malaria control and elimination management and programming. The district is an appropriate conduit between the technical and strategic oversight of the national level program and the communities at risk of malaria [15]. Placing the district at the center of CQPI enables all levels of the system to tackle highly contextual challenges while improving staff motivation.

### CQPI pilot studies

Although evidence on program management improvement approaches for malaria control and elimination is scarce, CQPI was piloted at the district-level in Eswatini (2016-17), Zimbabwe (2016-18) and Namibia (2019-20). The intervention design and methodologies evolved and were refined over the time period of three pilot studies. However, certain core elements were common to all three pilots. The learnings from the pilot program form the backbone of the LEAD Framework [22]. Table 2 provides an overview of the methodologies employed in the pilots, limitations of these studies and the practical lessons learned with respect to the implementation of CQPI.

**Table 2:** Pilot studies methodologies, activities, data collection methods and analysis, limitations, implementation lessons

**Countries, Provinces and Districts**	**Methodologies** (See Table 1 for definitions and methods)	**Activities**	**Data Collection & Analysis**
**Eswatini (1 malaria season 2016-17)** Country-wide project	PPI exclusively	Pre-malaria season ‘system in the room’ workshops (c.40 participants) – challenge identification and formation of Task Team implementation subgroup, external expert inputs on malaria elimination; Coaching and facilitation support to individuals and teams; 3 x in-season Task Team workshops (c.12 participants) to develop and implement work plans; Post-malaria season ‘system in the room’ workshop – review outcomes and planning for next season (c.40 participants).	Workshop and Task Team participation evaluation tools Metrics for monitoring and evaluation of specific challenges developed in liaison with NMCP and font line staff (closest to the issues). Data collected and analysed by Task Team – aided by project team experts. Monitored through implementation country work plan. Results reported to sponsor via project team.
**Zimbabwe (3 malaria seasons 2016-19)** Matabeleland South Beitbridge Gwanda Matapos Matabeleland North Binga Bubi Hwange Lupane Nkayi Tsholotsho Umgaza Midlands Chirumhanzu Kwekwe	PPI, QI, QM	**NB the following activities were repeated 3 x 2016-19)** Pre-malaria season ‘system in the room’ workshops (c.40-50 participants) – challenge identification and formation of Task Team implementation subgroups (12 in total), external expert inputs on malaria elimination; University certified training in CQPI (6 graduates); Coaching and facilitation support to individuals and teams; 3 x in-season Task Team (TT) workshops (c.12 participants) for each of the 12 districts (i.e., 12 TTs x 3) to develop and implement work plans; Post-malaria season ‘system in the room’ workshop – review outcomes and planning for next season (c.40-50 participants).	Workshop and Task Team participation evaluation tools Metrics for monitoring and evaluation of specific challenges developed in liaison with NMCP and font line staff (closest to the issues). Data collected and analysed by Task Team – aided by project team experts. Monitored through the 12 district-level implementation work plans. Results reported to sponsor via project team.
**Namibia (1 malaria season 2019-20)** Kavango East Kavango West	PPI, QI, QM, in the form of the LEAD Framework	Pre-malaria season ‘system in the room’ workshop (c.50 participants) – challenge identification and formation of 2 x Task Team implementation subgroups (8 per district team – 16 total), external expert inputs on malaria elimination; University certified training in CQPI (12 graduates); Coaching and facilitation support to individuals and teams; 6 x in-season Task Team workshops for the 2 districts to develop and implement work plans; Post-malaria season ‘system in the room’ workshop – review outcomes and planning for next season (c.50 participants).	Workshop and Task Team participation evaluation tools Metrics for monitoring and evaluation of specific challenges developed in liaison with NMCP and font line staff (closest to the issues). Data collected and analysed by Task Team – aided by project team experts. Monitored through the 2 district-level implementation work plans. Results reported to sponsor via project team.
**Limitations** - The impact of external influences on the program and outcomes was not assessed (e.g., co-investment by other agencies such as the United States Agency for International Development/President’s Malaria Initiative and/or the Global Fund to Fight AIDS, TB, and Malaria may have indirectly impacted some pilot studies results). - Neither experimental nor quasi-experimental design was employed. Control districts were not included as part of the pilots from which routine data could be collected as a comparison to intervention districts. Therefore we cannot say that the CQPI intervention was causal with improvement, only that in the observational pilot programs that CQPI is likely to have been the driver of improvement. - Project costs were relatively high in the design phase. With the training of local facilitators, costs decreased in later stages of implementation (e.g., graduates of a university certified training program in Zimbabwe were employed as consultants to assist with CQPI implementation in Namibia). - Limited evidence gathered for sustainability post-project due to limited funding and sustainability planning.			
**Implementation: key lessons** - It is imperative to negotiate and secure authorization for CQPI intervention at ministry level (e.g., official endorsement by NMCP director). NMCP-level participation in key CQPI events, such as, inception workshops and provincial review workshops is highly desirable as this can facilitate top level buy-in and support. In one of the pilots, the NMCP director changed mid-stream and the new role holder withdrew support for CQPI. This severely compromised the process and prevented further outcomes being achieved. - Active (authorized) participation of senior provincial staff in CQPI activities, e.g., Provincial Medical Directors (PMDs) attending and contributing to CQPI workshops and taking an active interest in the development and outcomes of district-level work plans. A supportive PMD often has the ability to mobilize the resources necessary to implement work plans. - Similarly, enrolment of senior district-level staff is critically important to successful implementation of CQPI. - The fuller the representation of the ‘system in the room’ (see Table 1 for definition) at key CQPI events, the better the chances of identifying and implementing ‘joined-up’ service delivery sollutions. Over the course of the three pilots, we learned that the involvement and buy-in of community leaders and influencers (e.g., faith leaders, traditional healers, etc.) impacted outcomes positively. - Devolvement of budgets to subnational level serves to improve implementation of solutions (enhances responsiveness of local actors to malaria challenges). Devolved budgets are planned in many countries as part of Universal Health Coverage plans.			

Table 3 provides a summary of the results for all three pilot studies. Results for Zimbabwe have been published elsewhere [21] and for Namibia will be forthcoming. Evidence of outcomes is encouraging, demonstrating the feasibility of improving productivity of district-level teams relatively easily and cheaply. The key driver of improvement is the increased ability of healthcare professionals to identify task and role-specific challenges in local contexts and work together to overcome them in collaboration with community stakeholders. By enabling in-depth analysis, problem solving and ownership by district offices and health facilities, CQPI built up an awareness of specific challenges and created an accountable process for action.

**Table 3:** Outcomes from CQPI pilots in malaria programs in Eswatini, Namibia and Zimbabwe [21]

Country	Year of implementation	Notable outcomes
Eswatini, nationwide	2016-2017	Improvements in the reporting of malaria cases by health facilities and increased collaboration between the malaria program, schools, and community organisations. It also led to improved communication between leaders within the NMCP.
Zimbabwe, 2 Districts	2016-2018	Increase in the availability of malaria registers from 83 to 93% (25/30 health facilities to 28/30 health facilties) , a reduction in artemisinin combination therapy stockouts from 22 to 6%, and an increase in the timeliness of case investigation within three days from 55 to 65% (65 cases investigated out of 119 reported to 821 cases investigated out of 1,265 cases reported). A second year resulted in a further improvement in the timeliness of case investigation to 92%, together with better interprovincial collaboration, and the initiation of meetings to harmonize surveillance.
Zimbabwe, 11 Disticts	2017-2018	In Matabeleland North, one year of implementation resulted in an increase in the administration of primaquine from 63% (90 cases treated/142 RDT positive cases) to 75% (76/101), an increase in slide examination rates from 81 to 89% (115 slides examined/142 RDT positive 142 cases to 90/101), an increase in fully investigated cases from 88% (125 cases fully investigated out of 142 RDT positive cases) to 98% (99 cases fully investigated out of 101 RDT positive cases), the development of a system to reduce stockouts of drugs and diagnostics that resulted in an improvement from 50 to 70% stock, and the increased disbursement of LLINS from 37 to 98% (14,535 to 38,499 out of 39,285 LLINs) by moving distribution centers closer to villages. In Midlands, operational improvements included an increase in the correct treatment of confirmed malaria cases from 93 to 100% in one district and an increase from 89 to 100% in another district and an improvement in case investigation rates from 80 to 100%. Qualitative results for this season in Matabeleland North, included: increased collaboration with partners involved in malaria activities and improvements in staff motivation and accountability. In Midlands province, outcomes included: improvements to data quality, completeness, and timeliness; increased community engagement activities; and improved communication, ownership, and teamwork. More importantly, participants across all provinces reported an increased ability to analyze problems, act on solutions, and measure performance.
Namibia, 2 Districts	2019-2020	40% increase in reporting (60% complete, timely reports to 100% (4131/4131) in both districts), a 32% average increase in cross-border reporting and tracing of malaria cases (41 to 79% (55/70) in Nankudu and 20 to 45% (41/91) in Rundu), and a 10% average increase in improved management of malaria cases (89 to 100% (2778/2778) in Nankudu and 89 to 98% (1326/1353) in Rundu), integration of malaria activities into the operational plans of local platforms, an elevated profile for malaria among other infectious diseases, and increased access to subnational resources, including vehicles, fuel, and radio spots. The programme was institutionalised into existing structures within the health system, and participants have integrated the relevant skills and approaches in their respective roles, providing evidence of sustainability beyond the programme period.

### Proposed changes in practice to improve malaria program management

The successful pilot of CQPI affords an opportunity to build a scalable, sustainable and effective health systems improvement model for the complex challenge of district-led malaria control and elimination. This framework should incorporate prioritized process and outcome indicators to guide the challenge areas, although the choice of activities and indicators for specific improvement should be made at the operational unit of delivery, proposed as the district-level, with inclusion of essential national program indicators if requested by the national level. The LEAD framework based on the CQPI pilots in Eswatini and Zimbabwe, and then tested in Namibia, is available as a tool for reference and for others to use [22].

**Fig. 1:**
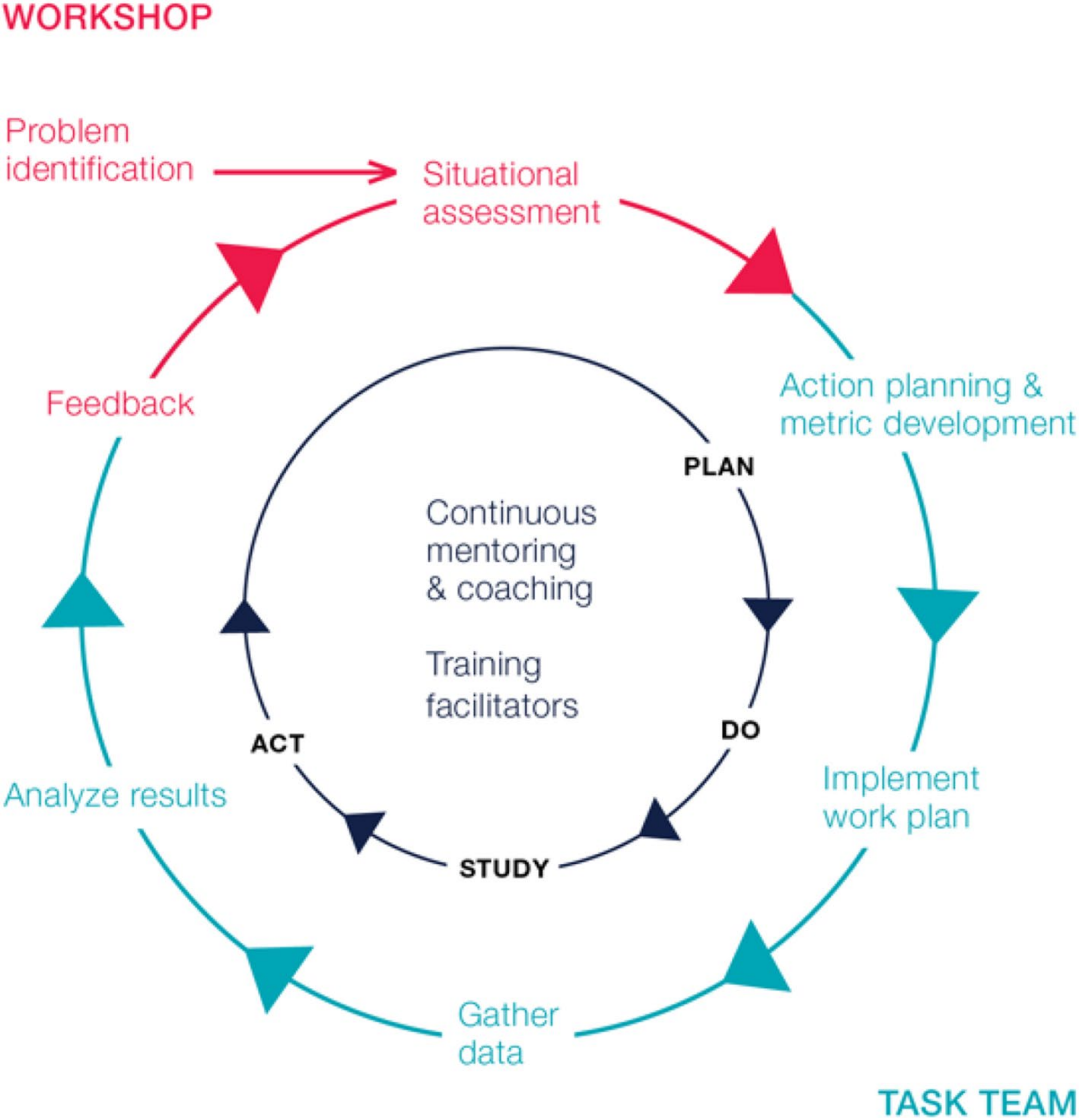
Participative Process Improvement Model for District and Provicincial Teams [21]. This figure depicts the annual PPI cycle, starting with an initial workshop consisting of the ‘system in the room’ at the top, where problems are identified and a situational assessment is conducted. Participants include representatives from national and provincial malaria and health leadership, district workers from cadres involved in delivering malaria activities and community representatives including local politicians, traditional healers etc. that should receive them. A prioritized list of problems are then transformed into a work plan with associated metrics by a self-selected multidisciplinary Task Team of 8-10 people. The Task Team implements the work plan, devising solutions to each challenge, gathers data, and analyzes results in a Plan-Do-Study-Act cycle, while also receiving continuous mentoring and coaching. At the same time, local facilitators are trained in how to lead the workshops and Task Team meetings. A follow-up workshop closes the loop, during which progress on problem-solving is fed back to the group, and the cycle begins again with the resolution of some problems and the addition of new problems

When implementing CQPI, people from across the vertical and horizontal layers of the system, including key community actors, were drawn together to focus attention on current challenges for implementation in districts, facilities and communities. Techniques such as root cause analysis, peer-led problem solving, and attentive listening were applied. Such methods enabled responsibilities to be identified, priorities agreed, improvement metrics established and a cross-functional taskforce – a smaller sub-grouping of the ‘system in the room’ (see Table 1 PPI definition) - selected on the basis of a representative staff ‘fit’ with the process improvement work that needed to be done. Regular structured reviews ensured that milestones were met, and further techniques were introduced as needed. The process described above should be repeated at least annually, to support continuous identification of new challenges and support for relevant initiatives to tackle them. When building PPI into malaria program strategies, the framework shown in Fig. 1 can be applied.

Expert facilitation of this workshop process is necessary to ensure a continuous focus on challenges and to prioritize and mitigate dynamics that could silence or marginalize perspectives that may offer crucial insight. These dynamics include (but are not limited to) effects of unequal status, gender, hierarchical position, resource-based power, and so forth. This expert facilitation can be ‘home grown’, as was the case for the CQPI pilot studies in Eswatini, Namibia and Zimbabwe, where malaria workers undertook an accredited 6-month training, and thereafter gradually took on facilitation, first supporting and then replacing the external facilitators. We recommend on-going peer-supervision for facilitators, under the guidance of senior professionals, ideally located in the same country.

### Investment opportunity

The CQPI approach offers an inexpensive path to significant improvements to the delivery and effectiveness of malaria control tools. It also has the potential to strengthen the overall health system. Efforts to improve operational performance of health systems must no longer be neglected in place of “magic bullets” such as vaccines or gene drives. Technologies that improve collation and presentation of information, such as spatial decision support systems suggesting courses of action for workers are helpful [24], but without the skills to make decisions, identify organizational inefficiencies, and develop adaptive responses, “data for decision-making” efforts will fail [25]. Technology and information alone will not solve the malaria challenges faced by districts.

This gap between information and effective problem solving can only be bridged by engaged staff who are motivated by being part of a committed and well-supported team with shared objectives and clear lines of accountability. These are the necessary *organizational* conditions for successful application of technical improvements. Reassuring financiers that new technologies will actually be used and integrated into the health care process is critical. CQPI has been effective in filling this gap between information and effective problem solving, but it now needs further evidence to back it up before countries and financiers will invest at large scale.

The CQPI approach addresses several issues currently faced in the malaria space, including the need to create a platform for true community participation in the design and implementation of malaria control and elimination strategies, as well as the need to transition from malaria-centric to programmatic approaches targeting multiple disease areas. CQPI has the potential to engage varied private sector and traditional practitioners in the design and implementation of locally tailored strategies, addressing an unmet need [2].

### A path forward for policy makers, financing institutions, implementers and researchers

Malaria programs are in need of a paradigm shift; one that places effective management and efficient organization at the centre of efforts to achieve high quality care [2]. As countries progress towards malaria elimination, we anticipate the growing importance of insight from districts and communities to improve the delivery of malaria services. Results from Eswatini, Namibia and Zimbabwe suggest that improving program management will be scalable, relatively inexpensive, and effective. Furthermore, CQPI supports the movement towards Universal Health Coverage (UHC), enabling frontline workers to deliver high quality care in central locations and at the fringes of the system. In short, it provides the means to harvest some very ripe ‘low hanging fruit’. What follows are suggested next steps that key stakeholders should take.

#### Policy makers

QI and QM for health systems service delivery is already being supported by the WHO through the Department of Service Delivery and Safety and the new National Quality Policy and Strategy Handbook [24]. This unit at the World Health Organisation (WHO) should consider the inclusion of CQPI alongside the other quality improvement interventions. Were the WHO to endorse applications of CQPI it would increase accountability and buy-in from the frontlines, building capacity and strengthening the quality of delivery necessary for UHC. We also recommend that the WHO Global Malaria Programme integrate the recommendations in the Department of Service Delivery and Safety strategies. Delivery is a long recognized challenge by the WHO, and malaria CQPI modules should be developed and implemented.

#### Financing institutions

Financing is required to implement CQPI strategies [2]. Sources of funding could include domestic financing, donor assistance, or a combination thereof. We recommend commencing with donor assistance to build an evidence base on the effectiveness of these combined strategies and approaches, and ascertain their expected low cost of implementation at scale [21].

We recommend a two-stage approach to financing this shift. The first would entail support from major international donors. This would be a novel area of investment for major donors, such as the Global Fund to Fight AIDS, Tuberculosis and Malaria, United States Agency for International Development, and Foreign, Commonwealth and Development Office, United Kingdom. These donors would need to decide if such investments would be for health systems generally, or disease-specific interventions. If they are not yet ready to invest in CQPI, what further evidence is required to consider their implementation, and who is willing to fund learning-by-doing projects to provide evidence to governments and donors? [2]

Presumably international donors will recognize these methods and give them an opportunity for wider implementation, allowing for their value to be recognized. If this stage is reached, we recommend that donor funding be carefully withdrawn in place of domestic funding, a transition we anticipate to be challenging but could take place gradually [2].

#### Implementers and researchers

We suggest three strands of information gathering for CQPI to improve district malaria program management. First, what are the costs and benefits of a Malaria CQPI program when implemented at scale? Clearly, evidence of cost-efficiency and impact of such a program would be helpful for policy makers and financiers of health. Second, what are the comparative and synergistic effects of CQPI and new technical solutions for targeting and tailoring interventions, such as Spatial Decision Support Systems [25], in settings where progress has plateaued? Third, how does CQPI improve worker motivation and health service utilization? Undoubtedly, evidence-based answers to this question would help financiers understand the additional benefits of improving district-level health performance. We have tried to set out the opportunity that CQPI affords, but the longer we wait, the longer we stall along the path to malaria eradication.

## Conclusion

Management challenges are widely cited as a barrier to malaria control and elimination and eventual eradication, yet training and capacity building in this area is typically targeted at the national level. CQPI offers a means of building management capacity at the district-level, where it is most needed. Pilot studies have shown that CQPI is feasible and scalable at low cost, and has resulted in important quantitative and qualitative improvements in malaria programs in Eswatini, Namibia and Zimbabwe. CQPI solutions to improve the acquisition of timely and detailed malaria surveillance data to enable swift and site-specific operational responses are needed for all locations approaching malaria elimination. The malaria community must resolve the paradox that results from simultaneously knowing that there is a problem in service delivery yet not being willing to invest in solutions that target that problem.

## Data Availability

Data sharing is not applicable to this article as no datasets were generated or analysed for this article. The results shared in Table 2 are in the public domain for Zimbabwe [21]. Data for Namibia are submitted for publication and are available from the corresponding author. Data from Eswatini are not publicly available due to restrictions in permissions and may be available from the corresponding author on reasonable request.

## Abbreviations

AIDS: Acute Immunodeficiency Syndrome
CQPI: Combined Quality and Process Improvement
HIV: Human Immunodeficiency Virus
LEAD: Leadership and Engagement for improved Accountability and Delivery of Services Framework
PPI: Participative Process Improvement
QI: Quality improvement
QM: Quality management
UHC: Universal Health Coverage
WHO: World Health Organisation
